# Receptor mechanisms of PAF mediated lymphatic constriction in the canine forelimb

**DOI:** 10.1155/S0962935192000590

**Published:** 1992

**Authors:** D. E. Dobbins

**Affiliations:** Department of Physiology Uniformed Services University of the Health Sciences 4301 Jones Bridge Road Bethesda MD 20814-4799 USA

## Abstract

Platelet activating factor (PAF) is a potent inflammatory lipid. In this study we assessed the ability of PAF to impact lymphatic vessel function by altering prenodal lymphatic resistance. Intralymphatic PAF (7.47 × 10^−6^, 7.47 × 10^−5^ and 7.47 × 10^−4^ M) increased lymphatic perfusion pressure at the two highest infusion rates. PAF mediated lymphatic constriction was not altered by the intra-arterial infusion of phentolamine but was blocked by the intra-arterial infusion of the PAF receptor antagonist WEB 2170. These data indicate that in addition to PAF's effects on microvascular permeability, this agent may also impact the ability of the lymphatics to transport fluid through alterations in lymphatic smooth muscle tone. PAF mediated lymphatic constriction is not mediated by α-receptors but rather through PAF receptor mediated mechanism.


Mediators of Inflammation 1, 391–395 (1992)

### Receptor mechanisms of PAF mediated lymphatic constriction in the canine forelimb

D. E. Dobbins

Department of Physiology, Uniformed Services University of the Health Sciences, 4301 Jones Bridge Road, Bethesda, MD 20814-4799, USA

**PLATELET activating factor (PAF) is a potent inflammatory lipid. In this study we assessed the ability of PAF to impact lymphatic vessel function by altering prenodal lymphatic resistance. Intralymphatic PAF ($7.47 \times 10^{-6}$, $7.47 \times 10^{-5}$ and $7.47 \times 10^{-4}$ M) increased lymphatic perfusion pressure at the two highest infusion rates. PAF mediated lymphatic constriction was not altered by the intra-arterial infusion of phentolamine but was blocked by the intra-arterial infusion of the PAF receptor antagonist WEB 2170. These data indicate that in addition to PAF's effects on microvascular permeability, this agent may also impact the ability of the lymphatics to transport fluid through alterations in lymphatic smooth muscle tone. PAF mediated lymphatic constriction is not mediated by α-receptors but rather through PAF receptor mediated mechanism.**

**Key words:** Inflammatory lipids, Lymphatic perfusion, Lymphatic smooth muscle, Lymphatic resistance, Lymph vessel function, Oedema, Transvascular fluid flux

### Introduction

Platelet activating factor (PAF) was first identified as a biological mediator when its release was shown from sensitized rabbit basophils which had been stimulated by antigen.[1] This factor was subsequently identified chemically as an acetyl glyceryl ether of phosphotidylcholine which is produced by leukocytes, macrophages, basophils and platelets from a number of species (including man) during anaphylactoid reactions.[2,3] PAF is a potent anaphylactoid agent and its administration in experimental animals results in cytopenia for platelets and neutrophils, systemic hypotension, platelet aggregation and degranulation, acute respiratory distress and release of a number of vasoactive compounds including histamine, serotonin, slow reacting substance of anaphylaxis and leukotriene $B_4$. Evidence is also accumulating which suggests that PAF may play an important role in the inflammatory process. Vemulapalli *et al.*[4] have shown that the intravenous administration of bolus doses of PAF in the anaesthetized dog results in marked decreases in plasma volume, indicating a significant increase in vascular permeability and transvascular fluid flux. Inarrea *et al.*[5] have shown that the infusion of immune aggregates in the mouse induced an increase in vascular permeability as evidenced by a nearly 25% reduction in blood volume. The decrease in blood volume was closely paralleled by the amount of PAF recovered from the tissues. Furthermore, the study revealed that the release of PAF preceded the increase in vascular permeability. Bjork *et al.*[6] have examined the effects of PAF on the microcirculation of the hamster cheek pouch *in situ*. They reported that the topical application of PAF caused constriction of small arteries and an increase in vascular permeability at the level of the postcapillary venule. We have previously shown that the intra-arterial infusion of PAF in the canine forelimb significantly increases microvascular permeability as indicated by increases in skin lymph flow and lymph protein concentration.[7] This action of PAF can be blocked by pretreatment of the forelimb with the $\beta_2$-receptor agonist terbutaline.

We have previously established that a number of endogenous vasoactive agents including adrenaline, noradrenaline, dopamine, prostanoids, putative inflammatory mediators and endothelin are capable of constricting prenodal lymph vessels in the canine forelimb.[8–12] In the current study, we assessed the ability of platelet activating factor to alter transvascular fluid flux by impacting the lymphatic system through alterations in lymphatic vessel resistance. In addition, we have assessed the involvement of lymphatic α-receptors and PAF receptors in the observed PAF mediated lymphatic constriction.

### Materials and Methods

Adult mongrel dogs of either sex were anaesthetized with sodium pentobarbital (35 mg/kg i.v. and supplemented as needed), intubated and ventilated with room air. Small incisions were made in the skin of the right forelimb and the brachial

© 1992 Rapid Communications of Oxford Ltd artery, a small side branch of the brachial artery and a skin small vein and artery in the paw were isolated. The forelimb was perfused at constant arterial inflow via the brachial artery with blood obtained from a cannulated femoral artery. Forelimb perfusion pressure was measured from the side branch of the brachial artery and skin small vein and artery pressures were measured on the dorsal and ventral surfaces of the paw respectively. Systemic arterial pressure was measured by inserting a catheter into the brachial artery and advancing it into the aorta. A catheter was inserted into the left external jugular vein and advanced to the level of the right atrium for measurement of central venous pressure. A prenodal lymph vessel was cannulated in the direction of normal lymph flow with a PE 10 catheter. The lymph vessel was perfused at constant flow at a volume flow rate of 0.034 ml/min with a perfusate which was the supernatant of a 1:1 mixture of autologous arterial blood and heparinized Krebs solution. Lymphatic perfusion pressure was measured from the perfusion system at a point upstream to the point of cannulation of the prenodal lymph vessel. A three-way stopcock, which had been modified such that all three ports were confluent, allowed for measurement of lymphatic perfusion pressure and for the lymphatic to be perfused with either control perfusate or perfusate containing PAF.[8]

The protocol was as follows: in all experiments, the lymph vessel was perfused with control perfusate for a minimum of 15 min to ensure that all measured pressures had reached steady state values. The lymph vessel was then perfused with a perfusate containing platelet activating factor in a concentration of $7.47 \times 10^{-6}$, $7.47 \times 10^{-5}$ or $7.47 \times 10^{-4}$ M for a minimum of 15 min or until the peak response was obtained. The lymph vessel was then again perfused with control perfusate until all measured pressures returned to control values. Preliminary experiments with PAF indicated that the initial infusion significantly attenuated the effects seen with any subsequent infusion in the same animal, a trait that PAF shares with angiotensin II, histamine and neurokinin A, and thus the three dosages were tested in three separate series of animals to reveal the true potency of PAF as a lymphatic constrictor.

In the PAF and receptor antagonist experiments, following acquisition of control values, an intra-arterial infusion of phentolamine (400 $\mu$g/min) or WEB 2170 (4 $\mu$g/min) was begun. After 10 min of phentolamine infusion or 15 min of WEB 2170 infusion, PAF was infused intralymphatically at a concentration of $7.47 \times 10^{4}$ M and the infusion was continued for 15 min. The lymphatic was then infused with control perfusate until all measured pressures returned to control values.

All drugs were made up freshly daily in normal saline. Phentolamine mesylate and WEB 2170 were infused via a needle tipped catheter into the arterial supply to the forelimb with the use of a pressure independent infusion pump. The final dilutions of PAF from the stock solution were made by adding control perfusate such that the osmolarity and protein concentration of the final solution approximated normal lymph.[8]

All data were analysed with Student's $t$-test as modified for paired replicates. Pressures obtained immediately prior to beginning a manoeuvre were compared with the peak responses observed during that manoeuvre. A probability of $\leq 0.05$ was considered statistically significant.

### Results

The intralymphatic infusion of PAF at $7.47 \times 10^{-6}$, $7.47 \times 10^{-5}$ and $7.47 \times 10^{-4}$ M resulted in significant increases in lymphatic perfusion pressure at the two higher dosages (Fig. 1). Lymphatic perfusion pressure in approximately half of the animals was increased by the lowest dosage of PAF utilized but variability in the response prevented the small increase seen at this dosage from being statistically significant. These data indicate that the threshold dosage for significant lymphatic constriction with intralymphatic PAF is very near $7.47 \times 10^{-6}$ M. It should be remembered that these data were derived from three separate series of animals and therefore the differences in the control lymphatic perfusion pressures seen in Fig. 1 do not reflect an instability of the preparation with time but rather modest differences in control lymphatic resistance between different groups of animals. The intralymphatic infusion of PAF did not significantly affect systemic, forelimb perfusion, skin small artery, skin small vein or central venous pressures (Table 1) at any of the infusion rates employed in this study.

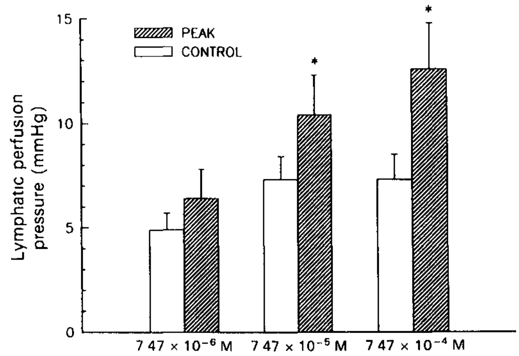

FIG. 1. The effects of the intralymphatic infusion of PAF on lymphatic perfusion pressure. $n = 8$, 8 and 9 in three separate groups of animals. * = $p \leq 0.05$, paired $t$.

**Table 1.** Effect of intralymphatic PAF infusion on vascular pressures. $n = 8$, 8 and 9 in each of three separate groups

| | $7.47 \times 10^{-6}$ | | $7.47 \times 10^{-5}$ | | $7.47 \times 10^{-4}$ | |
|---|---|---|---|---|---|---|
| | C | P | C | P | C | P |
| SYS | 131 ± 4.8 | 131 ± 4.5 | 131 ± 4.8 | 129 ± 5.6 | 123 ± 4.6 | 122 ± 4.5 |
| PERF | 117 ± 4.7 | 117 ± 4.8 | 115 ± 4.7 | 115 ± 5.8 | 121 ± 6.3 | 123 ± 7.3 |
| SSA | 85 ± 6.5 | 87 ± 6.3 | 85 ± 5.6 | 85 ± 6.4 | 93 ± 7.8 | 96 ± 8.5 |
| SSV | 9 ± 0.8 | 9 ± 0.8 | 11 ± 0.8 | 11 ± 0.9 | 10 ± 0.5 | 10 ± 0.5 |
| CV | 4 ± 0.2 | 4 ± 0.2 | 4 ± 0.4 | 4 ± 0.4 | 4 ± 0.5 | 3 ± 0.4 |

SYS = mean systemic, PERF = forelimb perfusion, SSA = skin small artery, SSV = skin small vein, CV = central venous. All pressures are means ± standard error of the mean expressed in mmHg. * = $p \leq 0.05$ Student's paired *t*. C = control, P = peak pressure.

The infusion of phentolamine intra-arterially at 400 μg/min did not significantly alter lymphatic perfusion pressure from its control value of 6.5 ± 1.2 mmHg (Fig. 2). As expected, phentolamine infusion did significantly decrease mean systemic arterial pressure and forelimb arterial pressures. Skin small vein pressure and central venous pressure were not significantly affected by phentolamine infusion (Table 2). The subsequent intralymphatic infusion of PAF at $7.47 \times 10^{-4}$ M significantly increased lymphatic perfusion pressure from a control value of 6.7 ± 1.3 mmHg to a peak pressure of 11.0 ± 1.9 mmHg (Fig. 2). This response is essentially identical to that seen with this dosage of PAF in the absence of α-receptor blockade (Fig. 1) and indicates that PAF mediated lymphatic constriction does not involve lymphatic α-receptors. We have previously shown that infusion of phentolamine at this dosage is capable of completely blocking either adrenaline or noradrenaline mediated lymphatic constriction.[8] The intralymphatic infusion of PAF during phentolamine infusion did not significantly affect vascular pressures (Table 2).

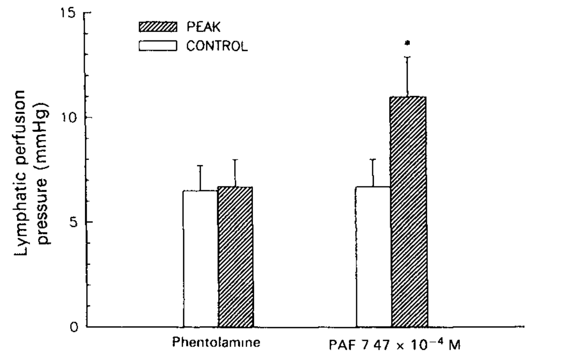

FIG. 2. The effects of intralymphatic PAF infusion during the intra-arterial infusion of phentolamine. $n = 6$. * = $p \leq 0.05$, paired *t*.

**Table 2.** Effect of intralymphatic PAF infusion during the intra-arterial infusion of phentolamine on vascular pressures

| | Phentolamine 400 μg/min | | PAF $7.47 \times 10^{-4}$ molar | |
|---|---|---|---|---|
| | C | P | C | P |
| SYS | 125 ± 2.6 | 117* ± 1.9 | 116 ± 2.3 | 105* ± 2.7 |
| PERF | 114 ± 3.9 | 91* ± 1.3 | 91 ± 1.5 | 87 ± 1.2 |
| SSA | 80 ± 7.3 | 58* ± 4.6 | 57 ± 5.0 | 53 ± 4.5 |
| SSV | 10 ± 0.6 | 10 ± 0.6 | 10 ± 0.6 | 10 ± 0.6 |
| CV | 4 ± 0.4 | 4 ± 0.4 | 4 ± 0.3 | 4 ± 0.3 |

SYS = mean systemic, PERF = forelimb perfusion, SSA = skin small artery, SSV = skin small vein, CV = central venous. All pressures are means ± standard error of the mean expressed in mmHg. * = $p \leq 0.05$ Student's paired *t*. C = control, P = peak pressure.

Intra-arterial infusion of the PAF receptor antagonist WEB 2170 at 4 μg/min does not significantly alter lymphatic perfusion pressure (Fig. 3) nor does it significantly affect vascular pressures (Table 3). The subsequent intralymphatic infusion of PAF during the continued infusion of WEB 2170 was not able to significantly alter the lymphatic perfusion pressure (Fig. 3). As before, intralymphatic PAF did not alter vascular pressures (Table 3).

### Discussion

It has been reported previously that a wide array of endogenous vasoactive agents are capable of constricting prenodal lymphatic vessels in the

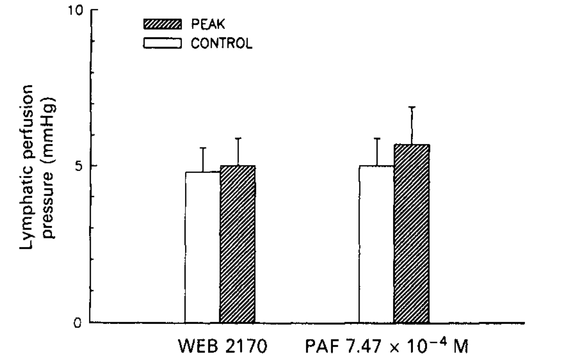

FIG. 3. The effects of intralymphatic PAF infusion during the intra-arterial infusion of the PAF receptor antagonist WEB 2170. $n = 6$. * = $p \leq 0.05$, paired *t*.

**Table 3.** Effects of intralymphatic PAF infusion during the intra-arterial infusion of WEB 2170 on vascular pressures

| | WEB 2170 4 μg/min | | PAF 7.47 x $10^{-4}$ molar | |
|---|---|---|---|---|
| | C | P | C | P |
| SYS | 130 ± 5.7 | 130 ± 6.0 | 130 ± 6.0 | 131 ± 4.6 |
| PERF | 117 ± 6.3 | 120 ± 5.9 | 120 ± 5.9 | 126 ± 4.8 |
| SSA | 83 ± 9.2 | 86 ± 10 | 86 ± 10 | 91 ± 8.3 |
| SSV | 8 ± 0.4 | 8 ± 0.4 | 8 ± 0.4 | 8 ± 0.5 |
| CV | 4 ± 0.2 | 4 ± 0.2 | 4 ± 0.2 | 4 ± 0.2 |

SYS = mean systemic, PERF = forelimb perfusion, SSA = skin small artery, SSV = skin small vein, CV = central venous. All pressures are means ± standard error of the mean expressed in mmHg. * = $p \leq 0.05$ Student's paired *t*. C = control, P = peak pressure.

canine forelimb. These agents include the catecholamines (adrenaline, noradrenaline and dopamine), endothelin, prostanoids, acetylcholine, bradykinin, histamine, serotonin and neurokinin A.[8–13] The results of the current study clearly demonstrate that PAF is likewise a member of the pool of endogenous vasoactive agents which can cause prenodal lymphatic constriction. Previous work has revealed that the threshold concentrations of these agents which are required to produce lymphatic constriction varies from the extremely potent endothelin ($10^{-10}$–$10^{-9}$ M) to markedly less potent agents such as prostaglandin $E_1$ and arachidonic acid ($10^{-4}$–$10^{-3}$ M). The results of the current experiments indicate that the threshold concentration of intralymphatic PAF is very near the lowest dosage used (7.47 x $10^{-6}$ M). Although the increase in lymphatic perfusion pressure seen with this dosage is not statistically different when viewed as a mean of eight animals, lymphatic perfusion pressure did increase in five of the eight animals. Thus it would appear that PAF is a moderately potent constrictor of lymphatic vessels with a threshold concentration similar to that of prostaglandin $F_{2\alpha}$ or the $\alpha_1$-receptor agonist phenylephrine and the $\alpha_2$-receptor agonist α-methylnorepinephrine.[10,14]

In the PAF and phentolamine experiments, the intralymphatic infusion of PAF during phentolamine resulted in an increase in lymphatic perfusion pressure which was not different from that which was seen with this same dosage of PAF in the non-α-receptor blocked preparation (Fig. 1). It has previously been shown[14] that the intra-arterial infusion of phentolamine at this dosage is capable of completely blocking either adrenaline or noradrenaline mediated lymphatic constriction. Therefore, this data would indicate that the lymphatic constriction produced by the intralymphatic administration of PAF is not mediated through lymphatic α-receptors although it has already been shown that the prenodal lymphatic vessels of the canine forelimb contain both $\alpha_1$- and $\alpha_2$-receptors.[14]

The intralymphatic infusion of PAF during the intra-arterial infusion of the specific PAF receptor antagonist WEB 2170[15–17] did not result in any significant alterations in lymphatic perfusion pressure. These data therefore indicate that the lymphatic constriction produced by PAF is mediated through lymphatic PAF receptor mechanisms. These data are in agreement with previous work indicating the effectiveness of specific PAF receptor antagonists in blocking PAF mediated vascular and cellular (platelet and neutrophil aggregation) actions. Felix *et al.*[18] showed that an intracoronary infusion of the PAF antagonist WEB 2086 antagonized PAF mediated coronary constriction in a dose-dependent manner. Ma *et al.*[15] reported that WEB 2170 inhibited the coronary constriction and tissue injury associated with coronary occlusion and reperfusion in a feline model. Klee *et al.*[16] demonstrated that WEB 2170 significantly inhibited the formation of microembolisms of rat platelets induced by platelet activating factor, whereas Heuer[17] reported that WEB 2170 inhibited PAF induced aggregation of human platelets *in vitro* but did not significantly affect the aggregation of platelets produced by ADP, adrenaline, serotonin, collagen or arachidonic acid. Additionally, he showed that WEB 2170 inhibited PAF induced platelet aggregation in the guinea-pig *in vivo*. Furthermore, the pretreatment of guinea-pigs with WEB 2170 either orally or intravenously completely protected the animals from the bronchoconstriction and systemic hypotension produced by intravenous infusion of PAF. In this respect, WEB 2170 displayed a significantly greater potency than WEB 2086. Thus WEB 2170 appears to be a highly specific and potent PAF receptor antagonist. The results of the current study indicate that the intra-arterial infusion of WEB 2170 is capable of completely blocking PAF mediated lymphatic constriction. These results suggest that the prenodal lymphatic vessels of the canine forelimb contain PAF receptors and it is through PAF receptor mechanisms that PAF produces increases in lymphatic smooth muscle tone.

Platelet activating factor has been implicated as a participant in a number of pathophysiological conditions including anaphylaxis, inflammation and oedema formation. It has been established that PAF increases microvascular permeability and transvascular fluid flux in a number of experimental models. In the canine forelimb, the increase in microvascular permeability seen in the skin circulation can be blocked by the intra-arterial infusion of the $\beta_2$-receptor agonist terbutaline.[7] In this respect, PAF shares a commonality with histamine and bradykinin whose ability to increase microvasvcular permeability in the canine forelimb is likewise blocked by terbutaline pretreatment. Of interest, histamine, bradykinin and now PAF have also been shown to possess the ability to constrict prenodal lymphatic vessels, thus affecting the ability of these vessels to transport fluid. PAF appears to be more potent than either histamine or bradykinin in producing lymphatic constriction.[11] Thus it would appear that a variety of agents can impact fluid balance through interactions with the microvascular membrane to alter its permiselectivity and can also impact the ability of the lymphatic system to transport fluid back to the vasculature.

### References

1. Benveniste J, Henson PM, Cochrane CG. Leukocyte dependent histamine release from rabbit platelets: the role of IgE, basophils and a platelet activating factor. *J Exp Med* 1972; **136**: 1356–1377.
2. Benveniste J. Platelet activating factor, a new mediator of anaphylaxis and immune complex deposition from rabbit and human basophils. *Nature* 1974; **249**: 581.
3. Pinckard RN, Halonen M, McManus LM, Hanahan DJ. Immunopharmacology of acetyl glyceryl ether phosphorycholine (AGEPC). In: Lenfant C, Newball H, eds. *Immunopharmacology of the lung*. New York, Marcel Dekker Inc.; 1982.
4. Vemulapalli S, Chiu PJS, Barnett A. Cardiovascular and renal action of platelet activating factor in anesthetized dogs. *Hypertension* 1984; **6(4)**: 489–493.
5. Inarrea P, Alonson F, Sanchez-Crespo M. Platelet activating factor: An effector substance of the vasopermeability changes induced by infusion of immune aggregates in the mouse. *Immunopharmacology* 1983; **6**: 7–14.
6. Bjork J, Lindbom L, Gerdin B, Smedegard G, Arfors K-E, Benveniste J. Paf-acether (platelet-activating factor) increases microvascular permeability and affects endothelium-granulocyte interaction in microvascular beds. *Acta Physiol Scand* 1983; **119**: 305–308.
7. Dobbins DE, Buehn MJ, Dabney JM. The inflammatory actions of platelet activating factor are blocked by levorotatory terbutaline. *Microcirc Endoth and Lymphatics* 1990; **6**: 437–455.
8. Dabney JM, Buehn MJ, Dobbins DE. Constriction of lymphatics by catecholamines, carotid occlusion and hemorrhage. *Am J Physiol* 1988; **255**: H514–H524.
9. Dabney JM, Dobbins DE. Perfused prenodal lymphatic vessels in the canine forelimb constrict in response to dopamine. *J Physiol (London)* 1986; **371**: 83P.
10. Dabney JM, Buehn MJ, Dobbins DE. Perfused prenodal lymphatics are constricted by prostaglandins. *Am J Physiol* 1991; **260**: H1–H5.
11. Dobbins DE, Buehn MJ, Dabney JM. Constriction of perfused lymphatics by acetylcholine, bradykinin and histamine. *Microcirc Endoth and Lymphatics* 1990; **6**: 409–425.
12. Dobbins DE, Dabney JM. Endothelin-mediated constriction of prenodal lymphatic vessels in the canine forelimb. *Regulatory Peptides* 1991; **35**: 81–91.
13. Dobbins DE. Neuropeptide modulation of lymphatic smooth muscle tone in the canine forelimb. *Mediators of Inflammation* 1992; **1**: 241–246.
14. Dobbins DE. Catecholamine-mediated lymphatic constriction: involvement of both $\alpha_1$- and $\alpha_2$-adrenoreceptors. *Am J Physiol* 1992; **263**: H473–H478.
15. Ma XL, Weyrich AS, Krantz S, Lefer AM. Mechanisms of the cardioprotective actions of WEB-2170, bepafant, a platelet activating factor antagonist, in myocardial ischemia and reperfusion. *J Pharmacol Exp Ther* 1992; **260(3)**: 1229–1236.
16. Klee A, Schmid-Schonbein GW, Seiffge D. Effects of platelet activating factor on rat platelets *in vivo*. *Eur J Pharmacol* 1991; **209(3)**: 223–230.
17. Heuer HO. Inhibition of active anaphylaxis in mice and guinea-pigs by the new hetrazepinoic PAF antagonist bepafant (WEB 2170).
18. Felix SB, Steger A, Bauman G, Busch R, Ochsenfeld G, Berdel WE. Platelet-activating factor-induced coronary constriction in the isolated perfused guinea-pig heart and antagonistic effects of the PAF antagonist WEB 2086. *J Lipid Mediators* 1990; **2(1)**: 9–20.

ACKNOWLEDGEMENTS. This work was supported by USUHS grant number RO 76 DD. The author gratefully acknowledges the generous gift of WEB 2170 from Boehringer Ingelheim Pharmaceuticals Inc.

**Received 18 August 1992; accepted in revised form 22 September 1992**
